# Molecular Profiling of *KIT/PDGFRA*-Mutant and Wild-Type Gastrointestinal Stromal Tumors (GISTs) with Clinicopathological Correlation: An 18-Year Experience at a Tertiary Center in Kuwait

**DOI:** 10.3390/cancers16162907

**Published:** 2024-08-21

**Authors:** Rola H. Ali, Ahmad R. Alsaber, Asit K. Mohanty, Abdulsalam Alnajjar, Eiman M. A. Mohammed, Mona Alateeqi, Hiba Jama, Ammar Almarzooq, Noelle Benobaid, Zainab Alqallaf, Amir A. Ahmed, Shakir Bahzad, Mohammad Alkandari

**Affiliations:** 1 Department of Pathology, College of Medicine, Kuwait University, Safat 13110, Kuwait; 2 Histopathology Laboratory, Sabah Hospital, Sabah Medical District, Safat 13001, Kuwait; 3 Department of Management, College of Business and Economics, American University of Kuwait, Safat 13034, Kuwait; aalsaber@auk.edu.kw; 4 Department of Medical Oncology, Kuwait Cancer Center, Sabah Medical District, Safat 13001, Kuwait; asitmohanty4u@gmail.com (A.K.M.); alnajjaronc@gmail.com (A.A.); 5 Molecular Genetics Laboratory, Kuwait Cancer Center, Sabah Medical District, Safat 13001, Kuwait; eiman_khajah@hotmail.com (E.M.A.M.); alateeqimona@gmail.com (M.A.); hiba.jama79@gmail.com (H.J.); am2004mar@gmail.com (A.A.); noelle.benobaid@hotmail.com (N.B.); zainabmsq@hotmail.com (Z.A.); amiral6666@gmail.com (A.A.A.); sbahzad@moh.gov.kw (S.B.); 6 Histopathology Laboratory, Farwaniya Hospital, Sabah Al Nasser Area 92426, Kuwait; moh.alkandari@gmail.com

**Keywords:** gastrointestinal stromal tumor (GIST), *KIT*, platelet-derived growth factor receptor alpha (*PDGFRA*), wild-type GIST, progression

## Abstract

**Simple Summary:**

Although gastrointestinal stromal tumor (GIST) is a relatively rare mesenchymal neoplasm of the digestive tract, molecular advancements in GISTs over the past two decades have caused a paradigm shift in the field of precision oncology. GISTs are mainly driven by activating mutations in the *KIT* or *PDGFRA* oncogenes, rendering them sensitive to targeted therapies. However, there is a significant lack of data from Kuwait that needs to be addressed. We carried out this retrospective analysis of a cohort of 200 GIST patients at the Kuwait Cancer Center to provide much-needed insights into the genetic makeup and clinicopathological characteristics of our population. We detailed the mutational spectrum in *KIT* and *PDGFRA*, identified a small subgroup of wild-type tumors, and shed some light on the clinical implications. This study opens the doors for potential larger-scale, multi-institutional outcome studies in the Arabian Gulf region.

**Abstract:**

In gastrointestinal stromal tumors (GISTs), identifying prototypical mutations in the *KIT/PDGFRA* oncogenes, or in rare alternate genes, is essential for prognostication and predicting response to tyrosine kinase inhibitors. Conversely, wild-type GISTs (WT-GIST), which lack known mutations, have limited treatment options. Data on the mutational landscape of GISTs and their impact on disease progression are very limited in Kuwait. Using a targeted next-generation sequencing panel, we investigated the spectrum and frequency of *KIT*, *PDGFRA*, and RAS-pathway-related mutations in 95 out of 200 GISTs diagnosed at Kuwait Cancer Center from 2005 to 2023 and assessed their correlation with clinicopathological parameters. Among the 200 tumors (median age 55 years; 15–91), 54% originated in the stomach, 33% in the small bowel, 7% in the colorectum, 1.5% in the peritoneum, and 4.5% had an unknown primary site. Of the 95 molecularly profiled cases, 88% had a mutation: *KIT* (61%), *PDGFRA* (25%), *NF1* (2%), and one *NTRK1* rearrangement. Ten WT-GISTs were identified (stomach = 6, small bowel = 2, and colorectum = 2). WT-GISTs tended to be smaller (median 4.0 cm; 0.5–8.0) (*p* = 0.018), with mitosis ≤5/5 mm^2^, and were of lower risk (*p* = 0.019). *KIT* mutations were an adverse indicator of disease progression (*p* = 0.049), while wild-type status did not significantly impact progression (*p* = 0.934). The genetic landscape in this cohort mirrors that of global studies, but regional collaborations are needed to correlate outcomes with genetic variants.

## 1. Introduction

Gastrointestinal stromal tumor (GIST) is a distinctive oncologic-molecular paradigm and a leading example of the utility of tyrosine kinase inhibitor (TKI)-targeted therapies. Although accounting for <1% of gastrointestinal neoplasms, GISTs are the most common mesenchymal tumors of the digestive tract [1]. They originate from the interstitial cells of Cajal or related stem cells in the gut wall [2], primarily arising in the stomach (50–60%), followed by the small bowel (30–35%) [3], and less frequently in the colorectum (5%), esophagus (<1%), and very rarely in the appendix and extra-visceral locations [4,5,6,7]. Their biological behavior is variable, ranging from indolent to highly aggressive, with risk stratification dependent on tumor size, location, and mitotic rate, which are the main prognostic parameters in localized disease [8].

GIST genotyping has become the cornerstone of clinical management, predicting biological behavior and response to TKIs, thereby transforming the treatment of both localized and metastatic disease [9,10,11]. GISTs are driven by oncogenic mutations in *KIT* (60–70%) [12,13] or platelet-derived growth factor receptor-alpha (*PDGFRA*) (10–15%) [14], with 10–15% of tumors lacking *KIT/PDGFRA* mutations, referred to as wild-type GISTs (WT-GIST). Generally, *KIT* mutations, particularly those in exons 11 and 9, tend to be more aggressive than *PDGFRA* mutants [10,15]; however, exon 11 mutations exhibit the highest sensitivity to imatinib TKI treatment [15,16]. In contrast, *KIT* exon 9 mutations confer decreased sensitivity to imatinib, warranting a higher dosage [17], while *PDGFRA* exon 18 D842V mutations confer primary (innate) resistance, which is currently targeted by next-generation TKIs such as Avapritinib [16,18]. Secondary (acquired) resistance to TKIs develops due to additional mutations involving the ATP-binding pocket of the kinase domain (encoded by exons 13 and 14) or the kinase activation loop (encoded by exons 17 and 18) [19,20,21,22]. Poor response to standard TKIs is also a problem in WT-GISTs [23,24] that harbor alternative oncogenic drivers, namely inactivating alterations of the succinate dehydrogenase (*SDH*) genes [25] or mutations in the RAS family genes, e.g. *BRAF* or *NF1* [26,27,28,29], as well as in GISTs that are quadruple wild-type for *KIT/PDGFRA/SDH/RAS* [30].

Data on GIST in the Kuwaiti population is scarce. In this study, we aimed to analyze and molecularly profile 95 cases from a cohort of 200 patients treated at the Kuwait Cancer Center over an 18-year period to gain insights into the genetic landscape, clinicopathological characteristics, and the impact of genotype on disease progression.

## 2. Materials and Methods

### 2.1. Clinicopathological Data

This study included GIST pathology specimens referred to the Kuwait Cancer Center from 2005 to 2023. A retrospective review of medical and histopathological records was conducted to collect clinicopathological data. Pathology slides were reviewed by two consultant pathologists. The pathological parameters included (as per the College of American Pathologists cancer reporting protocols) tumor location, size, histological phenotype, mitotic count, TNM stage, risk assessment category, and immunohistochemical expression of KIT, DOG1, and CD34. Additionally, evidence of metastasis was documented. The clinical data reviewed comprised stage, progression details, and information on TKI therapy.

### 2.2. Molecular Analysis Data

Next-generation sequencing (NGS) was performed on formalin-fixed paraffin-embedded (FFPE) tissues using the Oncomine Comprehensive Assay v3 (OCA v3), a targeted panel that covers 161 cancer-relevant genes (Thermo Fisher Scientific, Waltham, MA, USA) [31] (see Supplementary Table S1 for the gene list). DNA and RNA were extracted using the RecoverAll Total Nucleic Acid Isolation Kit (Thermo Fisher Scientific, Waltham, MA, USA), and the nucleic acid concentration was measured with a Qubit 3.0 Fluorometer. Library preparation was conducted manually following the manufacturer’s instructions with the Ion AmpliSeq Library Kit Plus (Thermo Fisher Scientific, Waltham, MA, USA). Sequencing was carried out on the Ion Torrent S5 XL platform (Thermo Fisher Scientific, Waltham, MA, USA). Data analysis was performed using the Ion Reporter™ Software (v.5.10). Sequence reads were aligned to the human genome assembly GRCh37/hg19, achieving a minimum of 10 million total mapped reads. Variant calling was conducted with a minor allele frequency (MAF) cutoff of 5% and an average mean depth of at least 200×. To ensure data quality, additional parameters were applied: base quality score (Q30 or higher), mapping quality (MQ ≥ 60), duplicate read removal (threshold of <5% duplicates), and uniformity (≥90%). Variants were filtered based on predefined quality thresholds, including read depth (minimum of 20 reads supporting the variant), allele balance (≥20%), and strand bias (≤10%). The final variant call set was annotated with clinical and functional information using databases such as ClinVar, COSMIC, and dbSNP. Potential pathogenicity was assessed using tools like SIFT and PolyPhen-2 for comprehensive variant interpretation. Detected somatic variants included single-nucleotide variants (SNV), small deletions (del), insertions (ins), insertions/deletions (indel), and selective copy number variants.

### 2.3. Statistical Analysis

Descriptive statistics were calculated for continuous variables, including mean, median, range, and standard deviation, and for categorical variables, which were summarized using frequencies and graphical representations. Clinicopathological parameters were evaluated using univariate analysis: Pearson’s Chi-squared test for categorical variables and a two-sample independent *t*-test for continuous variables. A *p*-value of less than 0.05 was considered statistically significant. The statistical analysis was conducted using JAMOVI Version 2.5.7.0.

## 3. Results

### 3.1. Clinicopathological Findings

A total of 200 GIST patients were identified in the pathology records from 2005 to 2023. The clinicopathological findings for this cohort are summarized in Table 1. The median age at diagnosis was 55 years (range 15–91), with a male-to-female ratio of 3:2. The median tumor size was 5.5 cm (range 0.4–25.0 cm); 24 patients (12%) had incidental GISTs detected during a sleeve gastrectomy, with tumors ≤2 cm. The most common primary site was the stomach (54%), followed by the small intestine (33%), colorectum (7%), and peritoneum (1.5%), with nine tumors (4.5%) were of unknown primary site. Among colorectal GISTs, which are rare, the distribution was as follows: rectum (*n* = 8; 57%), left colon (*n* = 3), transverse colon (*n* = 1), and colon not otherwise specified (*n* = 2). The median size of colorectal tumors was 6.5 cm (range 0.5–24 cm). Extra-visceral peritoneal tumors (*n* = 3), known as extra-gastrointestinal stromal tumors (EGISTs), were larger with a median of 16 cm (range 11–24 cm).

Although spindle-cell morphology was distributed everywhere, it was predominant in the small bowel and colorectum, while epithelioid tumors were clustered in the stomach (18/23; 78%). High-risk tumors accounted for 100% of EGISTs, 70% of colorectal GISTs, 39% of small bowel GISTs, and 15% of gastric GISTs. No-risk to low-risk tumors were predominantly found in the stomach, comprising 70% of these cases. Regarding focality, four cases had synchronous tumors, including one associated with neurofibromatosis type 1 (NF1) (case #83). Immunohistochemical analysis revealed the expression of KIT, DOG1, and CD34 in 96.7%, 97.9%, and 80.2% of cases, respectively.

### 3.2. Genotype Analysis

#### 3.2.1. Overall Frequencies

Out of 200 tumors, 95 (47.5%) were sequenced, primarily from imatinib-naive primary tumor tissues (87%) and less frequently from metastatic tumor tissues (13%). NGS was conducted on fifty-five gastric tumors, twenty-seven small bowel tumors, seven colorectal tumors, two EGISTs, and four tumors of the unknown primary site. Overall, *KIT/PDGFRA* mutations were detected in 82 cases (86%), with *KIT* mutations present in 61% and *PDGFRA* mutations in 25%. Thirteen cases (14%) were wild-type for *KIT/PDGFRA* based on our NGS panel (Table 2). Annotated genetic variants and their allelic frequencies in the tumor DNA samples are detailed in Supplementary Table S2.

#### 3.2.2. *KIT/PDGFRA*-Mutant GISTs

**Figure 1:**
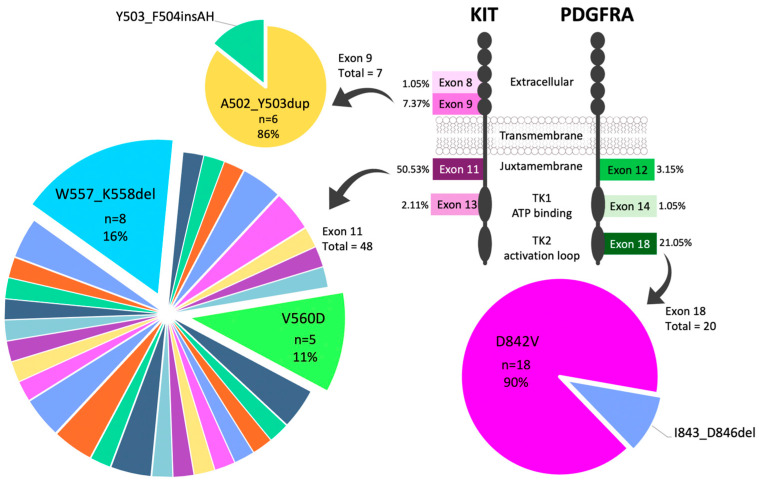
Frequencies of *KIT* and *PDGFRA* mutations (*n* = 95). *KIT* exon 11 mutations are heterogeneous, with W557_K558del being the most common. *PDGFRA* exon 18 and *KIT* exon 9 show a predominance of one variant each: D842V and A502_Y503dup, respectively. TK1 = Tyrosine kinase domain 1; TK2 = Tyrosine kinase domain 2.

In the *KIT*-mutant subgroup (*n* = 58), there were forty-eight mutations in exon 11 (encoding the juxtamembrane domain of the receptor), seven in exon 9 (extracellular Ig-like domain D5), two in exon 13 (ATP-binding pocket of the kinase domain), and one in exon 8 (extracellular domain) (Figure 1). The most common mutation type was in-frame deletions (40%), followed by SNVs (29%), insertions (17%), and indels (14%). 

In the *PDGFRA*-mutant subgroup (*n* = 24), there were twenty mutations in exon 18 (tyrosine kinase domain), three in exon 12 (juxtamembrane domain), and one in exon 14 (ATP-binding pocket of the kinase domain) (Figure 1). In contrast to *KIT* mutations, the great majority of *PDGFRA* mutations were SNVs (*n* = 21; 88%) (*p* < 0.001).

**Figure 2:**
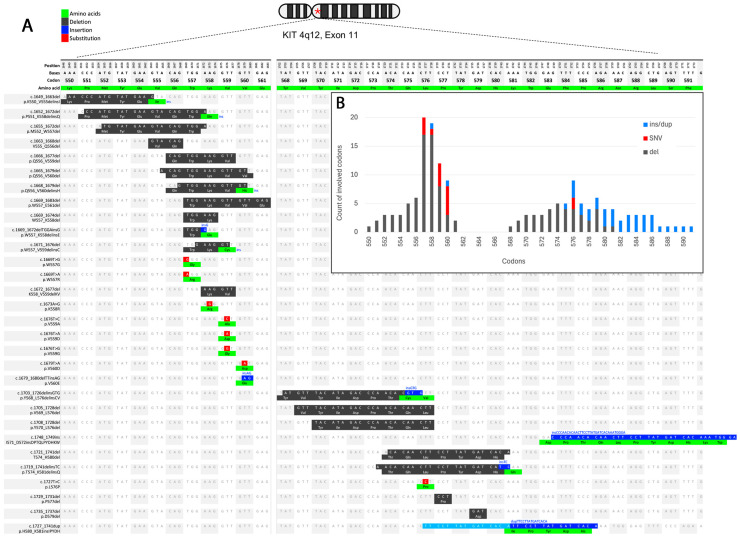
Genomic and amino-acid sequences of *KIT* exon 11 mutations. (**A**) Codon positions. (**B**) Frequency of codons involved in mutation. * *KIT* 4q12 locus.

*KIT* exon 11 mutations were diverse, spanning codons 550–561 and 568–591 (Figure 2). The most common variant was W557_K558del, accounting for 16% of exon 11 mutations. Among SNVs, the V560D substitution was the most frequent. The A502_Y503dup mutation was the most common in exon 9 (6/7 cases). For exon 13, the K642E variant was found in 2/2 cases. Additionally, a secondary (acquired) mutation, V654A, was identified in exon 13 following neoadjuvant imatinib treatment (case #22).

**Figure 3:**
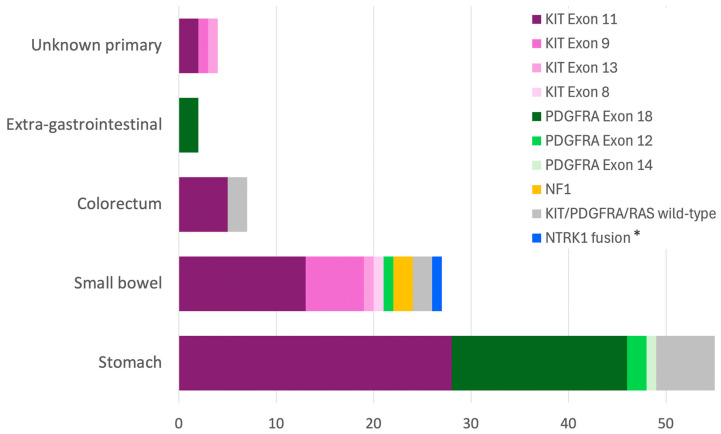
Distribution of molecular alterations based on gastrointestinal locations (*n* = 95). * *NTRK*-fused spindle cell neoplasms are currently classified as a separate entity.

For *PDGFRA*, the D842V substitution in exon 18 was the most prevalent, found in 75% of all *PDGFRA* cases. While most *PDGFRA* mutants were located in the stomach (87.5%), *KIT* mutants were observed throughout the gastrointestinal tract (*p* < 0.001) (Figure 3). *PDGFRA* D842V mutations were also detected in both EGISTs included in this study (cases #75 and 76). 

Within the stomach, *KIT* and *PDGFRA* mutations accounted for 51% and 38% of cases, respectively, with all *KIT* mutations involving exon 11. In the small bowel, *KIT* mutations accounted for 78% of cases, while *PDGFRA* was found in only one case. In this location, *KIT* mutations involved various exons: exon 11 (*n* = 13), exon 9 (*n* = 6), exon 13 (*n* = 1), and exon 8 (*n* = 1) (Figure 3). Exon 9 mutations were predominantly of the A502_Y503dup variant (5 of 6 cases), were exclusively found in the small bowel (with one exception in a tumor of unknown primary site), and were categorized as moderate- to high-risk. In the colorectum, only exon 11 *KIT* mutants were detected (5/7 cases).

**Figure 4:**
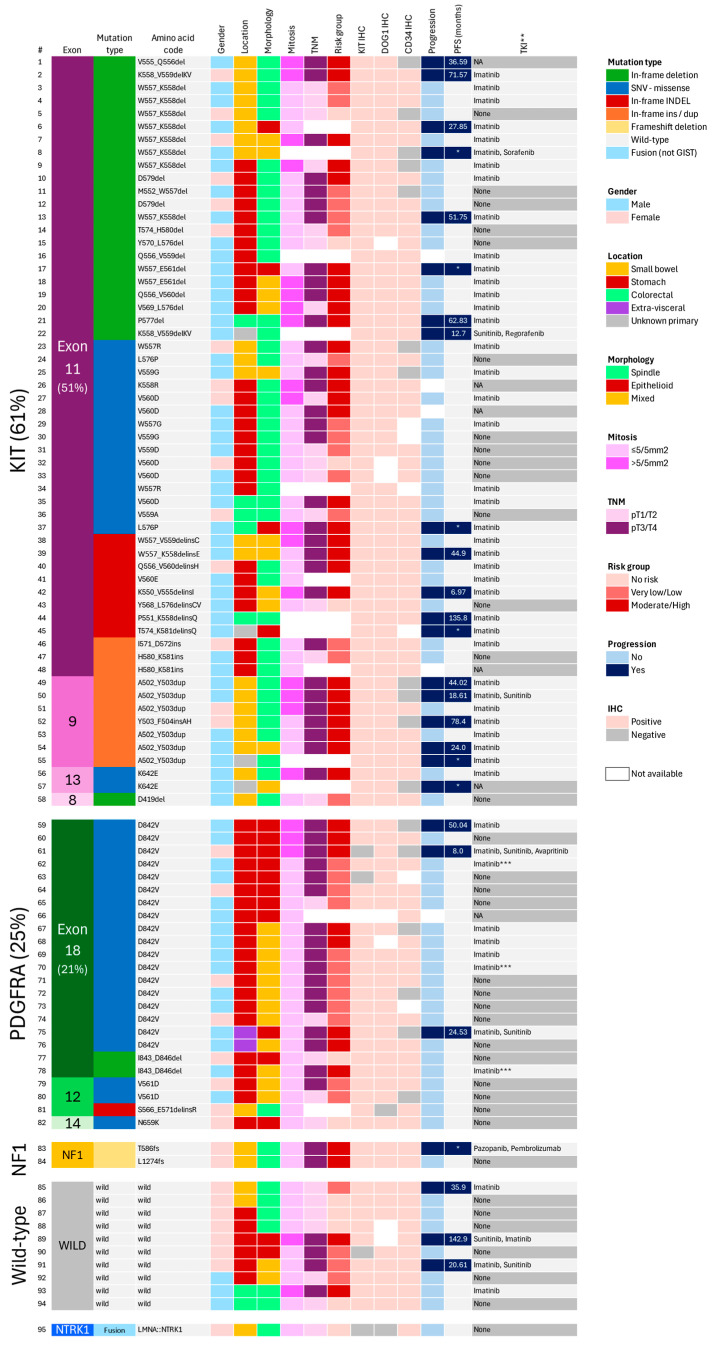
Graphical summary of molecular and clinicopathological findings (*n* = 95), with each row representing an individual patient. * Metastatic at diagnosis; ** Tyrosine kinase inhibitors, in adjuvant and/or metastatic setting; *** *PDGFRA*-specific TKIs not available; PFS = progression-free survival; IHC = immunohistochemistry; NA = not available.

*KIT* mutations were predominantly found in spindle-cell tumors (72%), while *PDGFRA* (both D842V and non-D842V) were primarily observed in purely epithelioid or mixed tumors (96%) (*p* < 0.001). A single spindle-cell *PDGFRA* mutant (S566_E571delinsR) was identified in the small bowel. No statistically significant differences were found concerning age, mean tumor size, mitotic index, TNM stage, pathological risk category, or KIT/DOG1 immunohistochemical expression. A summary of all cases with clinicopathological parameters is provided in Figure 4.

#### 3.2.3. *KIT/PDGFRA* Wild-Type GISTs

Among the thirteen *KIT/PDGFRA* wild-type GISTs, three harbored alternative molecular alternations: two had *NF1* mutations (including one case associated with known neurofibromatosis type 1 syndrome), and one had an *LMNA::NTRK1* gene fusion, leading to its reclassification as an “*NTRK*-rearranged spindle cell neoplasm” (previously reported [32]).

**Figure 5:**
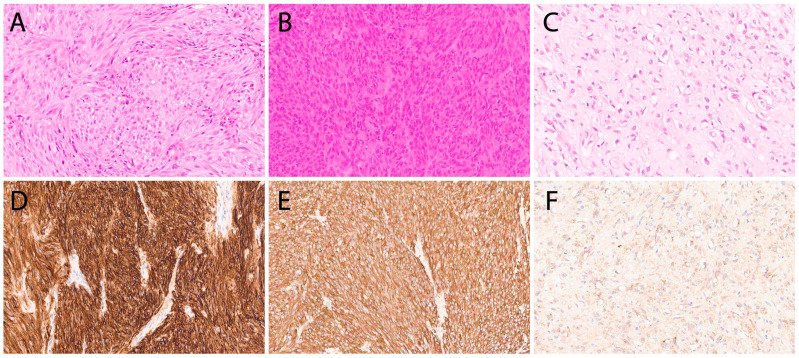
Histopathology of *KIT/PDGFRA* wild-type GISTs with corresponding KIT immunostaining. (**A**,**D**) *NF1*-mutant in the duodenum with spindle cell morphology and diffuse KIT expression in a known neurofibromatosis type 1 patient. (**B**,**E**) *KIT/PDGFRA/RAS* wild-type in the colon with spindle cell morphology and diffuse KIT expression. (**C**,**F**) *KIT/PDGFRA/RAS* wild-type in the stomach with epithelioid morphology and faint KIT expression. All are at 20× magnification.

Ten tumors were negative for mutations in *KIT*, *PDGFRA*, and RAS-pathway genes (*BRAF*, *RAS* isoforms, and *NF1*), and for all other 161 cancer-relevant genes that were included in our NGS panel. The median age of patients was 55 years, with a sex distribution of seven females and three males. The median tumor size was 4.0 cm, with four tumors ≤2 cm (range 0.5–8 cm). WT-GISTs were smaller in size compared to mutant GISTs (*p* = 0.018). The primary locations were the stomach (six cases), small bowel (two cases), and colorectum (two cases). Histological patterns included spindle cell (six cases), epithelioid (two cases), and mixed (two cases) (Figure 5). Mitotic counts were predominantly ≤5/5 mm^2^ (in eight cases). WT-GISTs appeared to be of lower risk (*p* = 0.019), with two of ten classified as high-risk. Immunohistochemically, all 10 cases expressed either KIT, DOG1, or both. SDH deficiency testing was not available.

#### 3.2.4. Disease Progression

Disease progression was defined as the first occurrence of loco-regional recurrence or metastasis detected through imaging or pathological examination. Clinical follow-up was available for 90 patients (range: 2 months–19 years; median: 4.4 years), of which 29% (*n* = 26) experienced disease progression, manifesting as either local recurrence (*n* = 3) or intra-abdominal/liver metastasis (*n* = 23). Seven tumors were metastatic at diagnosis. Univariate analysis revealed that progression was more common in small bowel locations (*p* = 0.001), larger tumors (*p* = 0.002), TNM stage pT3/T4 (*p* = 0.002), mitotic counts >5/5 mm^2^ (*p* = 0.004), and moderate- to high-risk categories (*p* < 0.001) (Table 3). Progression was observed in nineteen (35%) *KIT*-mutants (exon 11= 13; exon 9 = 5; exon 13 = 1) and in three (13%) *PDGFRA*-mutants. *KIT*-mutants had a higher likelihood of disease progression compared to *PDGFRA*-mutants (*p* = 0.049). Furthermore, *KIT*-mutants were more likely to present with advanced disease at diagnosis (10.7%), although this difference did not reach statistical significance. Three of the 10 WT-GISTs exhibited disease progression. There were no significant differences between *KIT* exon 11 and exon 9 mutations (*p* = 0.0815), deletions, and SNVs (*p* = 0.181) or between WT-GISTs and non-WT-GISTs (*p* = 0.934). Using the Cox Proportional Hazard model, no significant differences were detected between *KIT* and *PDGFRA* mutants or between WT-GISTs and mutants (*p* = 0.796 and *p* = 0.16, respectively).

## 4. Discussion

A substantial body of literature discusses the prognostic significance of mutational alterations in GISTs; however, the genetic landscape of GISTs and its impact on disease progression have not been investigated in our population. In this study, we retrospectively evaluated a cohort of 200 GIST cases diagnosed at the Kuwait Cancer Center over an 18-year period, offering a detailed description of the mutational spectrum in 95 cases.

The overall clinicopathological characteristics of this cohort were largely consistent with those reported in the literature. Gastric tumors were the most common, representing over half of all GISTs (54%) [33]. As expected, pure epithelioid GISTs clustered in the stomach and were very rare elsewhere, while spindle cell histology was observed at all sites but predominated in the small bowel (75%) and colorectum (86%). Colorectal GISTs, which made up 7% of this study’s cases, included eight rectal and six colonic tumors (three in the left colon, one in the transverse colon, and two unspecified). Although colorectal GISTs may represent two distinct subgroups, they are often combined due to their rarity [5]. Colonic GISTs, a rare subgroup, have a slight predilection for the left colon, with a risk of progression associated with large tumor size and high mitotic rate, similar to non-colonic counterparts [6]. Consistent with some reports [34,35], most of our colorectal cases (70%) were high-risk and appeared to have a worse prognosis compared to gastric cases. In descending order, high-risk tumors in this study comprised 100% of EGISTs, 70% of colorectal GISTs, 39% of small bowel GISTs, and 15% of gastric GISTs. Conversely, 70% of gastric GISTs were classified as no-risk to low-risk [36].

The overall frequency of gene mutations in this study was 88%, with 61% in *KIT*, 25% in *PDGFRA*, and 2% in *NF1*. The spectrum of *KIT/PDGFRA* mutations mirrored findings in the literature. *KIT* exon 11 mutations (the most common) included heterogeneous deletions/insertions/SNVs clustering around codons 550–561 and 568–591 [9,33]. The W557_K558del deletion and D842V substitution were the most prevalent among *KIT* exon 11 mutations. The D842V substitution accounted for 75% of *PDGFRA* cases [37]. *KIT* mutants were distributed throughout the entire GI tract, while the vast majority of *PDGFRA* mutations were localized to the stomach (*p* < 0.001). *KIT* mutations were predominantly found in spindle-cell tumors [38], whereas *PDGFRA* mutations were more common in epithelioid and mixed tumors (*p* < 0.001) [39]. Among tumors with mixed histological patterns, mutations were evenly divided between *KIT* and *PDGFRA*.

Two EGIST cases with epithelioid and mixed histology were identified as D842V-mutants, similar to gastric GISTs [40]. EGISTs located in the omentum, mesentery, and retroperitoneum occur in <10% of cases [7,41,42] and may, in some instances, represent extramural GISTs that have lost their connection to the gut wall. Omental and mesenteric EGISTs are likely related to gastric and small intestinal origins, respectively [43]. The rectal tumors in this study harbored *KIT* exon 11 mutations, while both analyzed colonic cases were wild-type for *KIT/PDGFRA/RAS*.

Another recurrent but less common mutation was *KIT* A502_Y503dup in exon 9 (observed in six of seven cases). *KIT* exon 9 mutants were localized exclusively in the small bowel (except for one case of an unknown primary site) and were associated with spindle-cell morphology. Less common exon 9 variants have been reported in the literature, including mutations occurring in gastric and rectal locations [44]. All *KIT* exon 9 mutants in this study were categorized as moderate- to high-risk, with disease progression detected in five of seven cases. The exon 13 K642E substitution [20,45,46], a primary mutation identified in two cases, has been reported in familial cases [47,48,49,50]. In contrast, the exon 13 V654A substitution, which was superimposed on an exon 11 mutation following neoadjuvant imatinib in one case, is typically a secondary mutation associated with acquired imatinib resistance [19,20,51,52].

Interestingly, a rectal GIST from a 61-year-old male (case #35) had a somatic *KIT* variant (c.1679T>A, p.V560D) superimposed on a familial *BRCA1* variant (c.4721delT, p.Leu1574fs). The tumor, classified as stage pT3/moderate-risk, was incidentally discovered during screening for prostate cancer. To date, no clear association exists between *BRCA1/2* mutations and GIST, with only rare reports available, including a patient harboring simultaneous *BRCA2* and *KIT* germline mutations manifesting as breast cancer and multiple GISTs, respectively [53,54]. Another curious finding in the current study was a *KIT* Exon 11 mutant in the ileum (case #4) showing a hypermutated genomic signature and microsatellite instability (MSI), although high MSI status is not typically involved in GIST tumorigenesis [55,56]. Only a single known hereditary syndromic case was included in this study: an NF1 patient with synchronous small bowl GISTs and advanced disease. Hereditary predisposition to GIST is rare and usually due to germline variants in *KIT*, *PDGFRA*, *NF1*, or *SDH* [48,57].

*KIT/PDGFRA* WT-GISTs are a genomically heterogeneous group of neoplasms, representing 10–15% of all GISTs. They are divided into SDH-deficient GISTs, which are almost exclusively gastric [25,30,58,59], and SDH-competent GISTs harboring mutations in *NF1* or *BRAF/RAS* (collectively referred to as RAS-pathway mutant GISTs) [26,27,60,61]. Quadruple WT-GISTs lack abnormalities in *KIT*, *PDGFRA*, *SDH*, and RAS signaling pathways [62]. Our WT-GISTs lacked *KIT/PDGFRA/RAS* mutations, but their *SDH* status was unknown. These included six gastric tumors, four of which had an epithelioid/mixed histology, raising the possibility of SDH deficiency. WT-GISTs appeared to be smaller in size compared to mutant tumors (*p* = 0.018) and were classified in a lower-risk category (*p* = 0.019). Three of ten WT-GISTs showed disease progression. Accurate characterization of WT-GISTs is needed as they respond poorly to standard TKIs and may benefit from alternative treatment options [23]. The single *NTRK*-rearranged spindle cell neoplasm in this study was labeled as such, as it seems to represent a distinct entity [63].

Tumor size, mitotic count, and small bowel location were the most powerful predictors of disease progression in this study. Tumor genotype seemed to be an important prognostic variable, with *KIT*-mutants showing a higher tendency to develop disease progression on univariate analysis [36], yet the difference did not reach statistical significance in the Cox model. Studies have shown that *KIT* exon 11 deletions involving codons 557 and/or 558 and exon 9 mutations are adverse prognostic indicators [64,65,66,67,68], while *KIT* exon 11 substitutions and insertions seem to have a more favorable prognosis [69]. *PDGFRA* mutations are generally associated with a more favorable outcome, occurring in gastric tumors with low or no malignant potential [36]. In the current study, no significant differences existed among *KIT* exon 11 and 9, deletion variants and SNVs, or WT-GIST and non-WT-GISTs.

Occasional extra-abdominal metastases were noted, with two tumors metastasizing to bone—specifically, rib and sternum. The rib metastasis, accompanied by a soft tissue mass resembling a primary soft tissue sarcoma, originated from a *KIT* exon 11-mutated colorectal GIST that had a protracted course of nearly two decades and was preceded via liver metastasis. Bone and soft tissue metastasis were rare in two large pioneering studies by Miettinen et al. [33,70].

## 5. Conclusions

This study provides a detailed description of the genetic landscape of GISTs in our population with insights into the clinicopathological characteristics, which is essential for refining GIST precision oncology in Kuwait. There are several limitations in this study, mainly related to its retrospective nature, the relative rarity of GISTs, and missing clinical data. The Cox Proportional Hazard model did not reach a level of significance for progression-free survival assessment. Overall, survival was difficult to use as an endpoint, given the relatively long survival of GIST patients and loss of follow-up.

## Figures and Tables

**Table 1:** Overall characteristics of the cohort (*n* = 200).

Variable	*n* (%)
**Age at presentation**	
Mean (SD)	54.3 (13.1)
Range	15.0–91.0
**Sex**	
Male	120 (60.0%)
Female	80 (40.0%)
**Location of primary tumor**	
Stomach	108 (54.0%)
Small bowel	66 (33.0%)
Colorectal	14 (7.0%)
Extra-visceral	3 (1.5%)
Unknown primary	9 (4.5%)
**Size of primary tumor (cm)**	
≤2 (T1)	32 (19.5%)
>2–≤5 (T2)	42 (25.6%)
>5–≤10 (T3)	55 (33.5%)
>10 (T4)	35 (21.3%)
Not available	36
**Histological phenotype**	
Spindle	127 (64.5%)
Epithelioid	23 (11.7%)
Mixed	47 (23.9%)
Not available	3
**Mitosis**	
≤5/5 mm^2^	133 (75.1%)
>5/5 mm^2^	44 (24.9%)
Not available	23
**Risk assessment**	
No risk	32 (19.2%)
Very low	17 (10.2%)
Low	43 (25.7%)
Moderate	28 (16.8%)
High	47 (28.1%)
Not available	33
**Stage at presentation**	
Stage I (I, IA, IB)	92 (53.2%)
Stage II	25 (14.5%)
Stage III (IIIA, IIIB)	39 (22.5%)
Stage IVA	17 (9.8%)
Not available	27
**Small incidental GISTs**	
Yes	24 (12.0%)
No	176 (88.0%)
**Status at 1st pathology encounter**	
Localized	183 (91.5%)
Advanced	17 (8.5%)

**Table 2:** Mutation frequencies in molecularly tested GISTs (*n* = 95).

Gene	*n* (%)
**KIT**	**58 (61.05%)**
Exon 11	48 (50.53%)
Exon 9	7 (7.37%)
Exon 13	2 (2.11%)
Exon 8	1 (1.05%)
**PDGFRA**	**24 (25.26%)**
Exon 18	20 (21.05%)
Exon 12	3 (3.15%)
Exon 14	1 (1.05%)
**NF1**	2 (2.11%)
**KIT/PDGFRA/RAS wild-type** *	**10 (10.53%)**
**NTRK1 fusion** **	**1 (1.05%)**

* RAS pathway genes include *BRAF*, *RAS* isoforms, and *NF1*. ** *NTRK*-fused spindle cell neoplasms are currently classified as a separate entity.

**Table 3:** Univariate analysis of clinicopathological features and disease progression.

Variable	No Progression (*n* = 64)	Progression (*n* = 26)	Total (*n* = 90)	*p* Value
**Age**				0.304 ^a^
Mean (SD)	55.4 (13.6)	52.2 (13.0)	54.5 (13.4)	
**Gene type**				* 0.049 ^b^
KIT	35 (63.6%)	19 (86.4%)	54 (70.1%)	
PDGFRA	20 (36.4%)	3 (13.6%)	23 (29.9%)	
Other	9	4	13	
**Wild-type**				0.934 ^b^
Yes	7 (10.9%)	3 (11.5%)	10 (11.1%)	
No	57 (89.1%)	23 (88.5%)	80 (88.9%)	
**Location of primary tumor**				* 0.001 ^b^
Stomach	43 (67.2%)	7 (26.9%)	50 (55.6%)	
Small bowel	16 (25.0%)	11 (42.3%)	27 (30.0%)	
Colorectal	4 (6.2%)	3 (11.5%)	7 (7.8%)	
Extra-visceral	1 (1.6%)	1 (3.8%)	2 (2.2%)	
Unknown primary	0 (0.0%)	4 (15.4%)	4 (4.4%)	
**Size (cm)**				* 0.002 ^a^
Mean (SD)	6.6 (5.0)	11.3 (5.9)	7.7 (5.5)	
Range	0.4–25.0	5.0–24.0	0.4–25.0	
Not available	3	9	12	
**Histological phenotype**				0.119 ^b^
Epithelioid	8 (12.5%)	8 (30.8%)	16 (17.8%)	
Mixed	20 (31.2%)	6 (23.1%)	26 (28.9%)	
Spindle	36 (56.2%)	12 (46.2%)	48 (53.3%)	
**TNM stage**				* 0.002 ^b^
pT1/T2	27 (44.3%)	1 (5.3%)	28 (35.0%)	
pT3/T4	34 (55.7%)	18 (94.7%)	52 (65.0%)	
Not available	3	7	10	
**Mitosis**				* 0.004 ^b^
≤5/5 mm^2^	52 (82.5%)	10 (50.0%)	62 (74.7%)	
>5/5 mm^2^	11 (17.5%)	10 (50.0%)	21 (25.3%)	
Not available	1	6	7	
**Risk category**				* <0.001 ^b^
No risk	11 (18.0%)	0 (0.0%)	11 (13.8%)	
Very low/Low	28 (45.9%)	3 (15.8%)	31 (38.8%)	
Moderate/High	22 (36.1%)	16 (84.2%)	38 (47.5%)	
Not available	3	7	10	

^a^ Linear Model ANOVA. ^b^ Pearson’s Chi-squared test. * Indicates statistical significance.

## Data Availability

All data are contained within this article.

## Supplementary Materials

The following supporting information can be downloaded at: https://www.mdpi.com/article/10.3390/cancers16162907/s1, Table S1: Genes included in the Oncomine Comprehensive Assay panel; Table S2: Annotated genetic variants and their allelic frequency in the tumor DNA samples.

## References

[1] Rubin B.P., Heinrich M.C., Corless C.L. (2007). Gastrointestinal stromal tumour. Lancet.

[2] Kindblom L.G., Remotti H.E., Aldenborg F., Meis-Kindblom J.M. (1998). Gastrointestinal pacemaker cell tumor (GIPACT): Gastrointestinal stromal tumors show phenotypic characteristics of the interstitial cells of Cajal. Am. J. Pathol..

[3] Joensuu H., Hohenberger P., Corless C.L. (2013). Gastrointestinal stromal tumour. Lancet.

[4] Rossi S., Miceli R., Messerini L., Bearzi I., Mazzoleni G., Capella C., Arrigoni G., Sonzogni A., Sidoni A., Toffolatti L. (2011). Natural history of imatinib-naive GISTs: A retrospective analysis of 929 cases with long-term follow-up and development of a survival nomogram based on mitotic index and size as continuous variables. Am. J. Surg. Pathol..

[5] Kukar M., Kapil A., Papenfuss W., Groman A., Grobmyer S.R., Hochwald S.N. (2015). Gastrointestinal stromal tumors (GISTs) at uncommon locations: A large population based analysis. J. Surg. Oncol..

[6] Hu S., Alpert L., Cates J.M.M., Gonzalez R.S., Rare GIST Risk Stratification Group (2022). Gastrointestinal stromal tumors (GISTs) arising in uncommon locations: Clinicopathologic features and risk assessment of esophageal, colonic, and appendiceal GISTs. Mod. Pathol..

[7] Miettinen M., Monihan J.M., Sarlomo-Rikala M., Kovatich A.J., Carr N.J., Emory T.S., Sobin L.H. (1999). Gastrointestinal stromal tumors/smooth muscle tumors (GISTs) primary in the omentum and mesentery: Clinicopathologic and immunohistochemical study of 26 cases. Am. J. Surg. Pathol..

[8] WHO Classification of Tumours Editorial Board (2020). WHO Classification of Tumours Series: Soft Tissue and Bone Tumours.

[9] Lasota J., Miettinen M. (2008). Clinical significance of oncogenic KIT and PDGFRA mutations in gastrointestinal stromal tumours. Histopathology.

[10] Rossi S., Gasparotto D., Miceli R., Toffolatti L., Gallina G., Scaramel E., Marzotto A., Boscato E., Messerini L., Bearzi I. (2015). KIT, PDGFRA, and BRAF mutational spectrum impacts on the natural history of imatinib-naive localized GIST: A population-based study. Am. J. Surg. Pathol..

[11] Ishikawa T., Kanda T., Kameyama H., Wakai T. (2018). Neoadjuvant therapy for gastrointestinal stromal tumor. Transl. Gastroenterol. Hepatol..

[12] Hirota S., Isozaki K., Moriyama Y., Hashimoto K., Nishida T., Ishiguro S., Kawano K., Hanada M., Kurata A., Takeda M. (1998). Gain-of-function mutations of c-kit in human gastrointestinal stromal tumors. Science.

[13] Nakahara M., Isozaki K., Hirota S., Miyagawa J., Hase-Sawada N., Taniguchi M., Nishida T., Kanayama S., Kitamura Y., Shinomura Y. (1998). A novel gain-of-function mutation of c-kit gene in gastrointestinal stromal tumors. Gastroenterology.

[14] Heinrich M.C., Corless C.L., Duensing A., McGreevey L., Chen C.J., Joseph N., Singer S., Griffith D.J., Haley A., Town A. (2003). PDGFRA activating mutations in gastrointestinal stromal tumors. Science.

[15] Joensuu H., Wardelmann E., Sihto H., Eriksson M., Sundby Hall K., Reichardt A., Hartmann J.T., Pink D., Cameron S., Hohenberger P. (2017). Effect of KIT and PDGFRA mutations on survival in patients with gastrointestinal stromal tumors treated with adjuvant imatinib: An exploratory analysis of a randomized clinical trial. JAMA Oncol..

[16] Heinrich M.C., Corless C.L., Demetri G.D., Blanke C.D., von Mehren M., Joensuu H., McGreevey L.S., Chen C.J., Van den Abbeele A.D., Druker B.J. (2003). Kinase mutations and imatinib response in patients with metastatic gastrointestinal stromal tumor. J. Clin. Oncol..

[17] Debiec-Rychter M., Sciot R., Le Cesne A., Schlemmer M., Hohenberger P., van Oosterom A.T., Blay J.Y., Leyvraz S., Stul M., Casali P.G. (2006). KIT mutations and dose selection for imatinib in patients with advanced gastrointestinal stromal tumours. Eur. J. Cancer..

[18] Heinrich M.C., Jones R.L., von Mehren M., Schöffski P., Serrano C., Kang Y.K., Cassier P.A., Mir O., Eskens F., Tap W.D. (2020). Avapritinib in advanced PDGFRA D842V-mutant gastrointestinal stromal tumour (NAVIGATOR): A multicentre, open-label, phase 1 trial. Lancet Oncol..

[19] Antonescu C.R., Besmer P., Guo T., Arkun K., Hom G., Koryotowski B., Leversha M.A., Jeffrey P.D., Desantis D., Singer S. (2005). Acquired resistance to imatinib in gastrointestinal stromal tumor occurs through secondary gene mutation. Clin. Cancer Res..

[20] Heinrich M.C., Maki R.G., Corless C.L., Antonescu C.R., Harlow A., Griffith D., Town A., McKinley A., Ou W.B., Fletcher J.A. (2008). Primary and secondary kinase genotypes correlate with the biological and clinical activity of sunitinib in imatinib-resistant gastrointestinal stromal tumor. J. Clin. Oncol..

[21] Gounder M.M., Maki R.G. (2011). Molecular basis for primary and secondary tyrosine kinase inhibitor resistance in gastrointestinal stromal tumor. Cancer Chemother. Pharmacol..

[22] Wakai T., Kanda T., Hirota S., Ohashi A., Shirai Y., Hatakeyama K. (2004). Late resistance to imatinib therapy in a metastatic gastrointestinal stromal tumour is associated with a second KIT mutation. Br. J. Cancer.

[23] Miranda C., Nucifora M., Molinari F., Conca E., Anania M.C., Bordoni A., Saletti P., Mazzucchelli L., Pilotti S., Pierotti M.A. (2012). KRAS and BRAF mutations predict primary resistance to imatinib in gastrointestinal stromal tumors. Clin. Cancer Res..

[24] Casali P.G., Abecassis N., Aro H.T., Bauer S., Biagini R., Bielack S., Bonvalot S., Boukovinas I., Bovee J.V.M.G., Brodowicz T. (2018). Gastrointestinal stromal tumours: ESMO-EURACAN Clinical Practice Guidelines for diagnosis, treatment and follow-up. Ann. Oncol..

[25] Janeway K.A., Kim S.Y., Lodish M., Nosé V., Rustin P., Gaal J., Dahia P.L., Liegl B., Ball E.R., Raygada M. (2011). Defects in succinate dehydrogenase in gastrointestinal stromal tumors lacking KIT and PDGFRA mutations. Proc. Natl. Acad. Sci. USA.

[26] Agaram N.P., Wong G.C., Guo T., Maki R.G., Singer S., Dematteo R.P., Besmer P., Antonescu C.R. (2008). Novel V600E BRAF mutations in imatinib-naive and imatinib-resistant gastrointestinal stromal tumors. Genes Chromosomes Cancer.

[27] Belinsky M.G., Rink L., Cai K.Q., Capuzzi S.J., Hoang Y., Chien J., Godwin A.K., von Mehren M. (2015). Somatic loss of function mutations in neurofibromin 1 and MYC associated factor X genes identified by exome-wide sequencing in a wild-type GIST case. BMC Cancer.

[28] Nishida T., Tsujimoto M., Takahashi T., Hirota S., Blay J.Y., Wataya-Kaneda M. (2016). Gastrointestinal stromal tumors in Japanese patients with neurofibromatosis type I. J. Gastroenterol..

[29] Huss S., Pasternack H., Ihle M.A., Merkelbach-Bruse S., Heitkötter B., Hartmann W., Trautmann M., Gevensleben H., Büttner R., Schildhaus H.U. (2017). Clinicopathological and molecular features of a large cohort of gastrointestinal stromal tumors (GISTs) and review of the literature: BRAF mutations in KIT/PDGFRA wild-type GISTs are rare events. Hum. Pathol..

[30] Boikos S.A., Pappo A.S., Killian J.K., LaQuaglia M.P., Weldon C.B., George S., Trent J.C., von Mehren M., Wright J.A., Schiffman J.D. (2016). Molecular subtypes of KIT/PDGFRA wild-type gastrointestinal stromal tumors: A report from the National Institutes of Health Gastrointestinal Stromal Tumor Clinic. JAMA Oncol..

[31] Vestergaard L.K., Oliveira D.N.P., Poulsen T.S., Høgdall C.K., Høgdall E.V. (2021). Oncomine Comprehensive Assay v3 vs. Oncomine Comprehensive Assay Plus. Cancers.

[32] Rahim S., Alkhaldi S.S., Alasousi K., Ali R.H. (2022). Intestinal LMNA::NTRK1-fused spindle cell neoplasm with S100 and CD34 coexpression: A new case. BMJ Case Rep..

[33] Miettinen M., Sobin L.H., Lasota J. (2005). Gastrointestinal stromal tumors of the stomach: A clinicopathologic, immunohistochemical, and molecular genetic study of 1765 cases with long-term follow-up. Am. J. Surg. Pathol..

[34] Feng F., Tian Y., Liu Z., Xu G., Liu S., Guo M., Lian X., Fan D., Zhang H. (2016). Clinicopathological features and prognosis of colonic gastrointestinal stromal tumors: Evaluation of a pooled case series. Oncotarget.

[35] Liu Z., Sun Y., Li Y., Zhao J., Wu S., Meng Z., Wu H. (2019). Colonic gastrointestinal stromal tumor: A population-based analysis of incidence and survival. Gastroenterol. Res. Pract..

[36] Lasota J., Dansonka-Mieszkowska A., Sobin L.H., Miettinen M. (2004). A great majority of GISTs with PDGFRA mutations represent gastric tumors of low or no malignant potential. Lab. Investig..

[37] Rizzo A., Pantaleo M.A., Astolfi A., Indio V., Nannini M. (2021). The identity of PDGFRA D842V-mutant gastrointestinal stromal tumors (GIST). Cancers.

[38] Wardelmann E., Neidt I., Bierhoff E., Speidel N., Manegold C., Fischer H.P., Pfeifer U., Pietsch T. (2002). c-kit mutations in gastrointestinal stromal tumors occur preferentially in the spindle rather than in the epithelioid cell variant. Mod. Pathol..

[39] Wardelmann E., Hrychyk A., Merkelbach-Bruse S., Pauls K., Goldstein J., Hohenberger P., Losen I., Manegold C., Büttner R., Pietsch T. (2004). Association of platelet-derived growth factor receptor alpha mutations with gastric primary site and epithelioid or mixed cell morphology in gastrointestinal stromal tumors. J. Mol. Diagn..

[40] Kanamori K., Yamagata Y., Honma Y., Date K., Wada T., Hayashi T., Otsuki S., Sekine S., Yoshikawa T., Katai H. (2020). Extra-gastrointestinal stromal tumor arising in the lesser omentum with a platelet-derived growth factor receptor alpha (PDGFRA) mutation: A case report and literature review. World J. Surg. Oncol..

[41] Reith J.D., Goldblum J.R., Lyles R.H., Weiss S.W. (2000). Extragastrointestinal (soft tissue) stromal tumors: An analysis of 48 cases with emphasis on histologic predictors of outcome. Mod. Pathol..

[42] Miettinen M., Felisiak-Golabek A., Wang Z., Inaguma S., Lasota J. (2017). GIST manifesting as a retroperitoneal tumor: Clinicopathologic immunohistochemical, and molecular genetic study of 112 cases. Am. J. Surg. Pathol..

[43] Agaimy A., Wünsch P.H. (2006). Gastrointestinal stromal tumours: A regular origin in the muscularis propria, but an extremely diverse gross presentation. A review of 200 cases to critically re-evaluate the concept of so-called extra-gastrointestinal stromal tumours. Langenbecks Arch. Surg..

[44] Künstlinger H., Huss S., Merkelbach-Bruse S., Binot E., Kleine M.A., Loeser H., Mittler J., Hartmann W., Hohenberger P., Reichardt P. (2013). Gastrointestinal stromal tumors with KIT exon 9 mutations: Update on genotype-phenotype correlation and validation of a high-resolution melting assay for mutational testing. Am. J. Surg. Pathol..

[45] Lux M.L., Rubin B.P., Biase T.L., Chen C.J., Maclure T., Demetri G., Xiao S., Singer S., Fletcher C.D., Fletcher J.A. (2000). KIT extracellular and kinase domain mutations in gastrointestinal stromal tumors. Am. J. Pathol..

[46] Lasota J., Wozniak A., Sarlomo-Rikala M., Rys J., Kordek R., Nassar A., Sobin L.H., Miettinen M. (2000). Mutations in exons 9 and 13 of KIT gene are rare events in gastrointestinal stromal tumors. A study of 200 cases. Am. J. Pathol..

[47] Graham J., Debiec-Rychter M., Corless C.L., Reid R., Davidson R., White J.D. (2007). Imatinib in the management of multiple gastrointestinal stromal tumors associated with a germline KIT K642E mutation. Arch. Pathol. Lab. Med..

[48] Bachet J.B., Landi B., Laurent-Puig P., Italiano A., Le Cesne A., Lévy P., Safar V., Duffaud F., Blay J.Y., Emile J.F. (2013). Diagnosis, prognosis and treatment of patients with gastrointestinal stromal tumour (GIST) and germline mutation of KIT exon 13. Eur. J. Cancer.

[49] Engin G., Eraslan S., Kayserili H., Kapran Y., Akman H., Akyuz A., Aykan N.F. (2017). Imatinib response of gastrointestinal stromal tumor patients with germline mutation on KIT exon 13: A family report. World J. Radiol..

[50] Peña-Irún A., Villa-Puente M., García-Espinosa R., Cavadas-López A. (2012). [Familial gastrointestinal stroma tumor due to mutation in exon 13 (K642E) of the KIT gene]. Med. Clin..

[51] Chen L.L., Trent J.C., Wu E.F., Fuller G.N., Ramdas L., Zhang W., Raymond A.K., Prieto V.G., Oyedeji C.O., Hunt K.K. (2004). A missense mutation in KIT kinase domain 1 correlates with imatinib resistance in gastrointestinal stromal tumors. Cancer Res..

[52] Serrano C., Mariño-Enríquez A., Tao D.L., Ketzer J., Eilers G., Zhu M., Yu C., Mannan A.M., Rubin B.P., Demetri G.D. (2019). Complementary activity of tyrosine kinase inhibitors against secondary kit mutations in imatinib-resistant gastrointestinal stromal tumours. Br. J. Cancer.

[53] Waisbren J., Uthe R., Siziopikou K., Kaklamani V. (2015). BRCA 1/2 gene mutation and gastrointestinal stromal tumours: A potential association. BMJ Case Rep..

[54] Sekido Y., Ohigashi S., Takahashi T., Hayashi N., Suzuki K., Hirota S. (2017). Familial gastrointestinal stromal tumor with germline KIT mutations accompanying hereditary breast and ovarian cancer syndrome. Anticancer Res..

[55] Campanella N.C., Scapulatempo-Neto C., Abrahão-Machado L.F., Torres De Oliveira A.T., Berardinelli G.N., Guimarães D.P., Reis R.M. (2017). Lack of microsatellite instability in gastrointestinal stromal tumors. Oncol. Lett..

[56] Park J., Sul H.J., Kim J.G. (2021). Rare occurrence of microsatellite instability in gastrointestinal stromal tumors. Medicina.

[57] Ricci R. (2016). Syndromic gastrointestinal stromal tumors. Hered. Cancer Clin. Pract..

[58] Miettinen M., Wang Z.F., Sarlomo-Rikala M., Osuch C., Rutkowski P., Lasota J. (2011). Succinate dehydrogenase-deficient GISTs: A clinicopathologic, immunohistochemical, and molecular genetic study of 66 gastric GISTs with predilection to young age. Am. J. Surg. Pathol..

[59] Killian J.K., Miettinen M., Walker R.L., Wang Y., Zhu Y.J., Waterfall J.J., Noyes N., Retnakumar P., Yang Z., Smith W.I. (2014). Recurrent epimutation of SDHC in gastrointestinal stromal tumors. Sci. Transl. Med..

[60] Agaimy A., Terracciano L.M., Dirnhofer S., Tornillo L., Foerster A., Hartmann A., Bihl M.P. (2009). V600E BRAF mutations are alternative early molecular events in a subset of KIT/PDGFRA wild-type gastrointestinal stromal tumours. J. Clin. Pathol..

[61] Gasparotto D., Rossi S., Polano M., Tamborini E., Lorenzetto E., Sbaraglia M., Mondello A., Massani M., Lamon S., Bracci R. (2017). Quadruple-Negative GIST Is a Sentinel for Unrecognized Neurofibromatosis Type 1 Syndrome. Clin. Cancer Res..

[62] Pantaleo M.A., Nannini M., Corless C.L., Heinrich M.C. (2015). Quadruple wild-type (WT) GIST: Defining the subset of GIST that lacks abnormalities of KIT, PDGFRA, SDH, or RAS signaling pathways. Cancer Med..

[63] Atiq M.A., Davis J.L., Hornick J.L., Dickson B.C., Fletcher C.D.M., Fletcher J.A., Folpe A.L., Mariño-Enríquez A. (2021). Mesenchymal tumors of the gastrointestinal tract with NTRK rearrangements: A clinicopathological, immunophenotypic, and molecular study of eight cases, emphasizing their distinction from gastrointestinal stromal tumor (GIST). Mod. Pathol..

[64] Wozniak A., Rutkowski P., Schöffski P., Ray-Coquard I., Hostein I., Schildhaus H.U., Le Cesne A., Bylina E., Limon J., Blay J.Y. (2014). Tumor genotype is an independent prognostic factor in primary gastrointestinal stromal tumors of gastric origin: A european multicenter analysis based on ConticaGIST. Clin. Cancer Res..

[65] Wardelmann E., Losen I., Hans V., Neidt I., Speidel N., Bierhoff E., Heinicke T., Pietsch T., Büttner R., Merkelbach-Bruse S. (2003). Deletion of Trp-557 and Lys-558 in the juxtamembrane domain of the c-kit protooncogene is associated with metastatic behavior of gastrointestinal stromal tumors. Int. J. Cancer..

[66] Martín J., Poveda A., Llombart-Bosch A., Ramos R., López-Guerrero J.A., García del Muro J., Maurel J., Calabuig S., Gutierrez A., González de Sande J.L. (2005). Deletions affecting codons 557-558 of the c-KIT gene indicate a poor prognosis in patients with completely resected gastrointestinal stromal tumors: A study by the Spanish Group for Sarcoma Research (GEIS). J. Clin. Oncol..

[67] Wang M., Xu J., Zhao W., Tu L., Qiu W., Wang C., Shen Y., Liu Q., Cao H. (2014). Prognostic value of mutational characteristics in gastrointestinal stromal tumors: A single-center experience in 275 cases. Med. Oncol..

[68] Kontogianni-Katsarou K., Dimitriadis E., Lariou C., Kairi-Vassilatou E., Pandis N., Kondi-Paphiti A. (2008). KIT exon 11 codon 557/558 deletion/insertion mutations define a subset of gastrointestinal stromal tumors with malignant potential. World J. Gastroenterol..

[69] Dematteo R.P., Gold J.S., Saran L., Gönen M., Liau K.H., Maki R.G., Singer S., Besmer P., Brennan M.F., Antonescu C.R. (2008). Tumor mitotic rate, size, and location independently predict recurrence after resection of primary gastrointestinal stromal tumor (GIST). Cancer.

[70] Miettinen M., Makhlouf H., Sobin L.H., Lasota J. (2006). Gastrointestinal stromal tumors of the jejunum and ileum: A clinicopathologic, immunohistochemical, and molecular genetic study of 906 cases before imatinib with long-term follow-up. Am. J. Surg. Pathol..

