# Supplementary material for: Molecular Profiling of KIT/PDGFRA-Mutant and Wild-Type Gastrointestinal Stromal Tumors (GISTs) with Clinicopathological Correlation: An 18-Year Experience at a Tertiary Center in Kuwait
Source: Cancers (Basel). 2024 Aug 21;16(16):2907. doi: 10.3390/cancers16162907 (PMC11352935; doi:10.3390/cancers16162907)
Supplement: Supplementary file 1 [file cancers-16-02907-s001.zip › cancers-3159199-supplementary.pdf]

Table S1: Genes included in the Oncomine Comprehensive Assay panel.

Hotspot genes				Full-length genes			Copy number genes		Gene fusions (inter- and intragenic)		
AKT1	ESR1	KIT	PDGFRB	ARID1A	FBXW7	PTEN	AKT1	FGFR4	AKT2	FGFR2	NUTM1
AKT2	EZH2	KNSTRN	PIK3CB	ATM	MLH1	RAD50	AKT2	FLT3	ALK	FGFR3	PDGFRA
AKT3	FGFR1	KRAS	PIK3CA	ATR	MRE11	RAD51	AKT3	IGF1R	AR	FGR	PDGFRB
ALK	FGFR2	MAGOH	PPP2R1A	ATRX	MSH6	RAD51B	ALK	KIT	AXL	FLT3	PIK3CA
AR	FGFR3	MAP2K1	PTPN11	BAP1	MSH2	RAD51C	AXL	KRAS	BRCA1	JAK2	PRKACA
ARAF	FGFR4	MAP2K2	RAC1	BRCA1	NBN	RAD51D	AR	MDM2	BRCA2	KRAS	PRKACB
AXL	FLT3	MAP2K4	RAF1	BRCA2	NF1	RNF43	BRAF	MDM4	BRAF	MDM4	PTEN
BRAF	FOXL2	MAPK1	RET	CDK12	NF2	RB1	CCND1	MET	CDKN2A	MET	PPARG
BTK	GATA2	MAX	RHEB	CDKN1B	NOTCH1	SETD2	CCND2	MYC	EGFR	MYB	RAD51B
CBL	GNA11	MDM4	RHOA	CDKN2A	NOTCH2	SLX4	CCND3	MYCL	ERBB2	MYBL1	RAF1
CCND1	GNAQ	MED12	ROS1	CDKN2B	NOTCH3	SMARCA4	CCNE1	MYCN	ERBB4	NF1	RB1
CDK4	GNAS	MET	SF3B1	CHEK1	PALB2	SMARCB1	CDK2	NTRK1	ERG	NOTCH1	RELA
CDK6	H3F3A	MTOR	SMAD4	CREBBP	PIK3R1	STK11	CDK4	NTRK2	ESR1	NOTCH4	RET
CHEK2	HIST1H3B	MYC	SMO	FANCA	PMS2	TP53	CDK6	NTRK3	ETV1	NRG1	ROS1
CSF1R	HNF1A	MYCN	SPOP	FANCD2	POLE	TSC1	EGFR	PDGFRA	ETV4	NTRK1	RSPO2
CTNNB1	HRAS	MYD88	SRC	FANCI	PTCH1	TSC2	ERBB2	PDGFRB	ETV5	NTRK2	RSPO3
DDR2	IDH1	NFE2L2	STAT3				ESR1	PIK3CB	FGFR1	NTRK3	TERT
EGFR	IDH2	NRAS	TERT				FGF19	PIK3CA			
ERBB2	JAK1	NTRK1	TOP1				FGF3	PPARG			
ERBB3	JAK2	NTRK2	U2AF1				FGFR1	RICTOR			
ERBB4	JAK3	NTRK3	XPO1				FGFR2	TERT			
ERCC2	KDR	PDGFRA					FGFR3				

Table S2: Annotated genetic variants and their allelic frequency in the tumor DNA samples.

	Key molecular alterations									Accompanying molecular alterations								
Case #	Gene	Exon	Variant type	Coding	Amino acid	Allelic frequency	Oncogenicity level	Clinical significance	COSMIC ID	Gene	Exon	Variant type	Coding	Amino acid	Allele frequency	Oncogenicity level	Clinical significance	COSMIC ID
1	KIT	Exon 11	In-frame del	c.1663_1668del	p.V555_Q556del	Not available	Likely oncogenic	Tier II - potential	COSM1201	_	_	_	_	_	_	_	_	_
2	KIT	Exon 11	In-frame del	c.1672_1677del	p.K558_V559delKV	44.31%	Oncogenic	Tier I - strong	COSM1235	_	_	_	_	_	_	_	_	_
3	KIT	Exon 11	In-frame del	c.1669_1674del	p.W557_K558del	19.87%	Oncogenic	Tier I - strong	COSM1210	_	_	_	_	_	_	_	_	_
4	KIT	Exon 11	In-frame del	c.1669_1674del	p.W557_K558del	24.17%	Oncogenic	Tier I - strong	COSM1210	Hypermutated genomic signature due to loss of function of mismatch repair (MMR) genes & microsatellite instability-high (MSI-H)								
5	KIT	Exon 11	In-frame del	c.1669_1674del	p.W557_K558del	Not available	Oncogenic	Tier I - strong	COSM1210	_	_	_	_	_	_	_	_	_
6	KIT	Exon 11	In-frame del	c.1669_1674del	p.W557_K558del	35.70%	Oncogenic	Tier I - strong	COSM1210	_	_	_	_	_	_	_	_	_
7	KIT	Exon 11	In-frame del	c.1669_1674del	p.W557_K558del	46.08%	Oncogenic	Tier I - strong	COSM1210	TERT	Locus 5p15.33	Amplification	_	_	Copy # 4.62	Likely oncogenic	Tier I - strong	_
8	KIT	Exon 11	In-frame del	c.1669_1674del	p.W557_K558del	Not available	Oncogenic	Tier I - strong	COSM1210	_	_	_	_	_	_	_	_	_
9	KIT	Exon 11	In-frame del	c.1669_1674del	p.W557_K558del	Not available	Oncogenic	Tier I - strong	COSM1210	_	_	_	_	_	_	_	_	_
10	KIT	Exon 11	In-frame del	c.1735_1737del	p.D579del	46.50%	Likely oncogenic	Tier II - potential	COSM1294	_	_	_	_	_	_	_	_	_
11	KIT	Exon 11	In-frame del	c.1655_1672del	p.M552_W557del	Not available	Likely oncogenic	Tier II - potential	COSM1180	_	_	_	_	_	_	_	_	_
12	KIT	Exon 11	In-frame del	c.1735_1737del	p.D579del	39.90%	Likely oncogenic	Tier II - potential	COSM1294	_	_	_	_	_	_	_	_	_
13	KIT	Exon 11	In-frame del	c.1669_1674del	p.W557_K558del	97.62%	Oncogenic	Tier I - strong	COSM1210	_	_	_	_	_	_	_	_	_
14	KIT	Exon 11	In-frame del	c.1721_1741del	p.T574_H580del	39.66%	Likely oncogenic	Tier I - strong	COSM24820	STK11	Not available	Frameshift del	c.369delG	p.M125fs	48.97%	Likely oncogenic	Not available	_
15	KIT	Exon 11	In-frame del	c.1708_1728del	p.Y570_L576del	23.64%	Likely oncogenic	Tier II - potential	COSM1285	PIK3CA	Exon 2	Missense- SNV	c.277C>T	p.R93W	49.27%	Oncogenic	Tier II - potential	COSM27493
16	KIT	Exon 11	In-frame del	c.1666_1677del	p.Q556_V559del	9.42%	Oncogenic	Tier I - strong	COSM36315	_	_	_	_	_	_	_	_	_
17	KIT	Exon 11	In-frame del	c.1669_1683del	p.W557_E561del	24.40%	Oncogenic	Tier I - strong	COSM1330	_	_	_	_	_	_	_	_	_
18	KIT	Exon 11	In-frame del	c.1669_1683del	p.W557_E561del	59.61%	Oncogenic	Tier I - strong	COSM1330	_	_	_	_	_	_	_	_	_
19	KIT	Exon 11	In-frame del	c.1665_1679del	p.Q556_V560del	53.13%	Likely oncogenic	Tier II - potential	COSM1198	_	_	_	_	_	_	_	_	_
20	KIT	Exon 11	In-frame del	c.1705_1728del	p.V569_L576del	46.07%	Likely oncogenic	Tier II - potential	COSM133635	_	_	_	_	_	_	_	_	_
21	KIT	Exon 11	In-frame del	c.1729_1731del	p.P577del	87.96%	Likely oncogenic	Tier II - potential	COSM1684822	_	_	_	_	_	_	_	_	_
22	KIT	Exon 11	In-frame del	c.1672_1677del	p.K558_V559delKV	50.03%	Oncogenic	Tier I - strong	COSM1235	TERT	Locus 5p15.33	Amplification	_	_	Copy # 5.82	Likely oncogenic	Tier I - strong	_
23	KIT	Exon 11	Missense- SNV	c.1669T>A	p.W557R	62.74%	Oncogenic	Tier I - strong	COSM1216	_	_	_	_	_	_	_	_	_
24	KIT	Exon 11	Missense- SNV	c.1727T>C	p.L576P	34.35%	Oncogenic	Tier I - strong	COSM1290	_	_	_	_	_	_	_	_	_
25	KIT	Exon 11	Missense- SNV	c.1676T>G	p.V559G	51.95%	Oncogenic	Tier II - potential	COSM1253	_	_	_	_	_	_	_	_	_
26	KIT	Exon 11	Missense- SNV	c.1673A>G	p.K558R	43.86%	Likely oncogenic	Tier II - potential	COSM29015	_	_	_	_	_	_	_	_	_
27	KIT	Exon 11	Missense- SNV	c.1679T>A	p.V560D	47.23%	Oncogenic	Tier I - strong	COSM1257	_	_	_	_	_	_	_	_	_
28	KIT	Exon 11	Missense- SNV	c.1679T>A	p.V560D	47.40%	Oncogenic	Tier I - strong	COSM1257	_	_	_	_	_	_	_	_	_
29	KIT	Exon 11	Missense- SNV	c.1669T>G	p.W557G	43.20%	Likely oncogenic	Tier II - potential	COSM1221	_	_	_	_	_	_	_	_	_
30	KIT	Exon 11	Missense- SNV	c.1676T>G	p.V559G	42.59%	Oncogenic	Tier II - potential	COSM1253	_	_	_	_	_	_	_	_	_
31	KIT	Exon 11	Missense- SNV	c.1676T>A	p.V559D	21.11%	Oncogenic	Tier II - potential	COSM1252	_	_	_	_	_	_	_	_	_
32	KIT	Exon 11	Missense- SNV	c.1679T>A	p.V560D	Not available	Oncogenic	Tier I - strong	COSM1257	_	_	_	_	_	_	_	_	_
33	KIT	Exon 11	Missense- SNV	c.1679T>A	p.V560D	59.89%	Oncogenic	Tier I - strong	COSM1257	HIST1H3B	Locus 6p22.2	Amplification	_	_	Copy # NA	Not available	Not available	_
34	KIT	Exon 11	Missense- SNV	c.1669T>A	p.W557R	22.81%	Oncogenic	Tier I - strong	COSM1216	CDKN1B	Locus 12p13.1	Amplification	_	_	Copy # 7.1	Uncertain	Tier III - uncertain	_
										CCND2	Locus 12p13.32	Amplification	_	_	Copy # 5.27	Uncertain	Tier III - uncertain	_
35	KIT	Exon 11	Missense- SNV	c.1679T>A	p.V560D	46.32%	Oncogenic	Tier I - strong	COSM1257	BRCA1	Not available	Frameshift del	c.4721delT	p.L1574fs	49.90%	Likely oncogenic	Not available	_
36	KIT	Exon 11	Missense- SNV	c.1676T>C	p.V559A	42.61%	Likely oncogenic	Tier I - strong	COSM1255	_	_	_	_	_	_	_	_	_
37	KIT	Exon 11	Missense- SNV	c.1727T>C	p.L576P	66.31%	Oncogenic	Tier I - strong	COSM1290	FBXW7	Not available	Nonsense- SNV	c.346G>T	p.E116*	62.71%	Likely oncogenic	Tier III - uncertain	_
38	KIT	Exon 11	INDEL	c.1671_1676del	p.W557_V559delinsC	26.22%	Likely oncogenic	Tier II - potential	COSM1233	_	_	_	_	_	_	_	_	_
39	KIT	Exon 11	INDEL	c.1669_1672delTGGAinsG	p.W557_K558delinsE	40.13%	Likely oncogenic	Tier II - potential	COSM24748	ERBB2	Exon 23	Missense- SNV	c.2790G>T	p.E930D	47.19%	Uncertain	Tier III - uncertain	COSV54065601
										NOTCH2	Exon 15	Missense- SNV	c.2373C>G	p.N791K	85.24%	Uncertain	Tier III - uncertain	COSV56700559
40	KIT	Exon 11	INDEL	c.1668_1679del	p.Q556_V560delinsH	41.89%	Likely oncogenic	Tier II - potential	COSM1213	RICTOR	Locus 5p13.1	Amplification	_	_	Copy # 6.1	Not available	Not available	_
41	KIT	Exon 11	INDEL	c.1679_1680delTTinsAG	p.V560E	42.42%	Likely oncogenic	Tier II - potential	COSM1333	_	_	_	_	_	_	_	_	_
42	KIT	Exon 11	INDEL	c.1649_1663del	p.K550_V555delinsI	48.81%	Oncogenic	Tier I - strong	COSM1164	_	_	_	_	_	_	_	_	_
43	KIT	Exon 11	INDEL	c.1703_1726delinsGTG	p.Y568_L576delinsCV	Not available	Likely oncogenic	Tier II - potential	COSM1279	_	_	_	_	_	_	_	_	_
44	KIT	Exon 11	INDEL	c.1652_1672del	p.P551_K558delinsQ	20.04%	Likely oncogenic	Tier II - potential	_	_	_	_	_	_	_	_	_	_
45	KIT	Exon 11	INDEL	c.1719_1741delinsTC	p.T574_K581delinsQ	Not available	Likely oncogenic	Tier II - potential	_	_	_	_	_	_	_	_	_	_
46	KIT	Exon 11	Ins / dup	c.1748_1749ins	p.I571_D572insDPTQLPYDHKW	25.85%	Likely oncogenic	Tier II - potential	COSM29430	_	_	_	_	_	_	_	_	_
47	KIT	Exon 11	Ins / dup	c.1727_1741dup	p.H580_K581insIPYDH	17.22%	Likely oncogenic	Tier I - strong	COSM51538	_	_	_	_	_	_	_	_	_
48	KIT	Exon 11	Ins / dup	c.1727_1741dup	p.H580_K581insIPYDH	Not available	Likely oncogenic	Tier I - strong	COSM51538	_	_	_	_	_	_	_	_	_
49	KIT	Exon 9	Ins / dup	c.1509_1510insGCCTAT	p.A502_Y503dup	18.13%	Oncogenic	Tier I - strong	_	_	_	_	_	_	_	_	_	_
50	KIT	Exon 9	Ins / dup	c.1509_1510insGCCTAT	p.A502_Y503dup	36.81%	Oncogenic	Tier I - strong	_	_	_	_	_	_	_	_	_	_
51	KIT	Exon 9	Ins / dup	c.1509_1510insGCCTAT	p.A502_Y503dup	41.34%	Oncogenic	Tier I - strong	_	_	_	_	_	_	_	_	_	_
52	KIT	Exon 9	Ins / dup	c.1509_1510insGCCCAT	p.Y503_F504insAH	47.39%	Oncogenic	Tier I - strong	_	_	_	_	_	_	_	_	_	_
53	KIT	Exon 9	Ins / dup	c.1509_1510insGCCTAT	p.A502_Y503dup	48.61%	Oncogenic	Tier I - strong	COSM1326	_	_	_	_	_	_	_	_	_
54	KIT	Exon 9	Ins / dup	c.1509_1510insGCCTAT	p.A502_Y503dup	31.96%	Oncogenic	Tier I - strong	COSM1326	_	_	_	_	_	_	_	_	_

55	KIT	Exon 9	Ins / dup	c.1509_1510insGCCTAT	p.A502_Y503dup	19.67%	Oncogenic	Tier I - strong	COSM1326	_	_	_	_	_	_	_	_	_
56	KIT	Exon 13	Missense- SNV	c.1924A>G	p.K642E	29.70%	Oncogenic	Tier II - potential	COSM1304	_	_	_	_	_	_	_	_	_
57	KIT	Exon 13	Missense- SNV	c.1924A>G	p.K642E	27.40%	Oncogenic	Tier II - potential	COSM1304	_	_	_	_	_	_	_	_	_
58	KIT	Exon 8	In-frame del	c.1255_1257del	p.D419del	38.12%	Likely oncogenic	Tier III - uncertain	COSM29014	_	_	_	_	_	_	_	_	_
59	PDGFRA	Exon 18	Missense- SNV	c.2525A>T	p.D842V	Not available	Oncogenic	Tier II - potential	COSM736	_	_	_	_	_	_	_	_	_
60	PDGFRA	Exon 18	Missense- SNV	c.2525A>T	p.D842V	38.37%	Oncogenic	Tier II - potential	COSM736	NOTCH3	Exon 11	Missense- SNV	c.1630C>T	p.R544C	Not available	Likely oncogenic	Tier III - uncertain	_
61	PDGFRA	Exon 18	Missense- SNV	c.2525A>T	p.D842V	18.78%	Oncogenic	Tier II - potential	COSM736	ALK	Locus 2p23.2	Amplification	_	_	Copy # 7.8	Likely oncogenic	Tier II - potential	_
										AR	Locus Xq12	Amplification	_	_	Copy # 9.2	Likely oncogenic	Tier II - potential	_
62	PDGFRA	Exon 18	Missense- SNV	c.2525A>T	p.D842V	Not available	Oncogenic	Tier II - potential	COSM736	_	_	_	_	_	_	_	_	_
63	PDGFRA	Exon 18	Missense- SNV	c.2525A>T	p.D842V	31.59%	Oncogenic	Tier II - potential	COSM736	_	_	_	_	_	_	_	_	_
64	PDGFRA	Exon 18	Missense- SNV	c.2525A>T	p.D842V	13.64%	Oncogenic	Tier II - potential	COSM736	_	_	_	_	_	_	_	_	_
65	PDGFRA	Exon 18	Missense- SNV	c.2525A>T	p.D842V	16.50%	Oncogenic	Tier II - potential	COSM736	_	_	_	_	_	_	_	_	_
66	PDGFRA	Exon 18	Missense- SNV	c.2525A>T	p.D842V	Not available	Oncogenic	Tier II - potential	COSM736	_	_	_	_	_	_	_	_	_
67	PDGFRA	Exon 18	Missense- SNV	c.2525A>T	p.D842V	33.70%	Oncogenic	Tier II - potential	COSM736	_	_	_	_	_	_	_	_	_
68	PDGFRA	Exon 18	Missense- SNV	c.2525A>T	p.D842V	15.93%	Oncogenic	Tier II - potential	COSM736	_	_	_	_	_	_	_	_	_
69	PDGFRA	Exon 18	Missense- SNV	c.2525A>T	p.D842V	35.28%	Oncogenic	Tier II - potential	COSM736	_	_	_	_	_	_	_	_	_
70	PDGFRA	Exon 18	Missense- SNV	c.2525A>T	p.D842V	25.35%	Oncogenic	Tier II - potential	COSM736	_	_	_	_	_	_	_	_	_
71	PDGFRA	Exon 18	Missense- SNV	c.2525A>T	p.D842V	17.13%	Oncogenic	Tier II - potential	COSM736	_	_	_	_	_	_	_	_	_
72	PDGFRA	Exon 18	Missense- SNV	c.2525A>T	p.D842V	22.32%	Oncogenic	Tier II - potential	COSM736	_	_	_	_	_	_	_	_	_
73	PDGFRA	Exon 18	Missense- SNV	c.2525A>T	p.D842V	36.99%	Oncogenic	Tier II - potential	COSM736	_	_	_	_	_	_	_	_	_
74	PDGFRA	Exon 18	Missense- SNV	c.2525A>T	p.D842V	31.44%	Oncogenic	Tier II - potential	COSM736	_	_	_	_	_	_	_	_	_
75	PDGFRA	Exon 18	Missense- SNV	c.2525A>T	p.D842V	34.06%	Oncogenic	Tier II - potential	COSM736	NOTCH1	Exon 24	Missense- SNV	c.3907C>T	p.R1303C	47.32%	Oncogenic	Tier II - potential	COSM6903650
76	PDGFRA	Exon 18	Missense- SNV	c.2525A>T	p.D842V	39.49%	Oncogenic	Tier II - potential	COSM736	_	_	_	_	_	_	_	_	_
77	PDGFRA	Exon 18	In-frame del	c.2527_2538del	p.I843_D846del	Not available	Likely oncogenic	Tier II - potential	COSM12400	_	_	_	_	_	_	_	_	_
78	PDGFRA	Exon 18	In-frame del	c.2527_2538del	p.I843_D846del	38.32%	Likely oncogenic	Tier II - potential	COSM12400	_	_	_	_	_	_	_	_	_
79	PDGFRA	Exon 12	Missense- SNV	c.1682T>A	p.V561D	34.92%	Oncogenic	Tier II - potential	COSM739	_	_	_	_	_	_	_	_	_
80	PDGFRA	Exon 12	Missense- SNV	c.1682T>A	p.V561D	19.45%	Oncogenic	Tier II - potential	COSM739	_	_	_	_	_	_	_	_	_
81	PDGFRA	Exon 12	INDEL	c.1698_1712del	p.S566_E571delinsR	6.72%	Likely oncogenic	Tier II - potential	COSM30546	_	_	_	_	_	_	_	_	_
82	PDGFRA	Exon 14	Missense- SNV	c.1977C>A	p.N659K	36.91%	Likely oncogenic	Tier II - potential	COSM22415	_	_	_	_	_	_	_	_	_
83	NF1	Exon16	Frameshift del	c.1756_1759delACTA	p.T586fs	81.94%	Likely oncogenic	Tier III - uncertain	COSM4770400	KRAS	Locus 12p12.1	Amplification	_	_	Copy # 8.7	Oncogenic	Tier II - potential	_
										RB1	Locus 13q14.2	Amplification	_	_	Copy # 8.2	Oncogenic	Tier II - potential	_
										SETD2	Not available	Frameshift del	c.629_630delA	p.E210fs	36.08%	Likely oncogenic	Not available	_
										CDK4	Locus 12q14.1	Amplification	_	_	Copy # 5.22	Oncogenic	Tier II - potential	_
84	NF1	Exon 28	Frameshift del	c.3821delT	p.L1274fs	78.45%	Likely oncogenic	Tier III - uncertain	_	_	_	_	_	_	_	_	_	_
85	wild	_	_	_	_	_	_	_	_	_	_	_	_	_	_	_	_	_
86	wild	_	_	_	_	_	_	_	_	_	_	_	_	_	_	_	_	_
87	wild	_	_	_	_	_	_	_	_	_	_	_	_	_	_	_	_	_
88	wild	_	_	_	_	_	_	_	_	_	_	_	_	_	_	_	_	_
89	wild	_	_	_	_	_	_	_	_	_	_	_	_	_	_	_	_	_
90	wild	_	_	_	_	_	_	_	_	_	_	_	_	_	_	_	_	_
91	wild	_	_	_	_	_	_	_	_	_	_	_	_	_	_	_	_	_
92	wild	_	_	_	_	_	_	_	_	_	_	_	_	_	_	_	_	_
93	wild	_	_	_	_	_	_	_	_	_	_	_	_	_	_	_	_	_
94	wild	_	_	_	_	_	_	_	_	_	_	_	_	_	_	_	_	_
95	NTRK1	Exon11	Gene fusion	LMNA::NTRK1 (exon2/exon11)	_	_	Oncogenic	Tier I - strong	COSF1653	_	_	_	_	_	_	_	_	_
